# Sex Estimation Through Orbital Measurements: A Machine Learning Approach for Forensic Science

**DOI:** 10.3390/diagnostics14242773

**Published:** 2024-12-10

**Authors:** George Triantafyllou, George G. Botis, Maria Piagkou, Konstantinos Papanastasiou, George Tsakotos, Ioannis Paschopoulos, George K. Matsopoulos, Stavroula Papadodima

**Affiliations:** 1 Department of Anatomy, School of Medicine, Faculty of Health Sciences, National and Kapodistrian University of Athens, 11 527 Goudi, Greece; georgerose406@gmail.com (G.T.); botis_g@biomed.ntua.gr (G.G.B.); mapian@med.uoa.gr (M.P.); gtsakotos@gmail.com (G.T.); johnpascho@gmail.com (I.P.); 2 Biomedical Engineering Laboratory, School of Electrical and Computer Engineering, National Technical University of Athens, 15 773 Zografou, Greece; papkonstpap@gmail.com (K.P.); gmatsopoulos@biomed.ntua.gr (G.K.M.); 3 Department of Forensic Medicine and Toxicology, School of Medicine, National and Kapodistrian University of Athens, 11 527 Goudi, Greece

**Keywords:** orbit, optic canal, optic foramen, orbital measurements, sex estimation, machine learning, anatomy, forensic science

## Abstract

Background: Sex estimation has been extensively investigated due to its importance for forensic science. Several anatomical structures of the human body have been used for this process. The human skull has important landmarks that can serve as reliable sex estimation predictors. Methods: In this study, orbital measurements from 92 dried skulls, comprising 35 males and 57 females, were utilized to develop a machine-learning-based classifier for sex estimation with potential applications in forensic science. The parameters evaluated included optic foramen height (OFH), optic foramen width (OFW), optic canal height (OCH), optic canal width (OCW), intraorbital distance (IOD), extraorbital distance (EOD), orbit height (OH), and orbit width (OW). Results: A Random Forest classifier was employed to analyze the data, achieving an overall test accuracy of 0.68. The model demonstrated a precision of 0.65, indicating a moderate level of false positives. The recall was 0.70, reflecting that 70% of the positive cases were correctly identified. The F1 score was calculated at 0.675, suggesting a balanced performance between precision and recall. The area under the curve (ROC AUC) score was also 0.72, indicating that the model can distinguish between classes. The most important features in the best subset were OW (0.2429), IOD (0.2059), EOD (0.1927), OFH (0.1798), and OFW (0.1787), highlighting their significant contributions to the model’s predictions. Conclusions: These findings suggest that orbital measurements could potentially serve as reliable predictors for automated sex estimation, contributing to advancements in forensic identification techniques

## 1. Introduction

Anatomy is an essential science with multiple implications in several medical specialties, such as neurosurgery, orthopedic surgery, abdominal surgery, otolaryngology, and forensics. Sex estimation has been the subject of extensive research for many years, while recently, the machine learning approach provided a new scope and expansion. Different anatomical structures of the human body have been used, such as the orbital measurements [1], skull shape [2], foramen magnum [3], long bones [4], and atlas vertebra [5]. The main clinical application of sex estimation is during forensic investigations of skeletal elements that may survive and be recovered [6]. Most commonly, the pelvis is the first choice for sex estimation [7]; however, different skull structures have been used because the skull is thicker and denser and less likely to be damaged over time [6].

The orbit has been investigated for its sexual dimorphism [1,6,8,9,10,11,12]. The bony orbit is one of the most complicated skull areas and is composed of four orbital walls (roof, floor, lateral, and medial) [13]. Several orbital measurements are performed, such as the orbit height (OH), orbit width (OW), infraorbital distance (IOD), and extraorbital distance (EOD). Nevertheless, the optic canal (OC) transmits the optic nerve (CN II) and the ophthalmic artery, while the optic foramen (OF) serves as the opening of the OC. Trauma and tumors frequently invade the orbital anatomy. Injury to the orbital floor leads to functional and aesthetic complications that affect the patient’s life quality. Knowledge of orbital floor morphometrics is paramount for adequate repair of the orbital region. Natsis et al. [13] proposed a simple method to evaluate the essential measurements of the orbital floor that can be used during reconstruction surgery. Nevertheless, anatomical landmarks of the medial orbital wall are important for surgeries in the orbit, such as fractures, reconstruction, tumor resection, and transsphenoidal sphenoidectomy [14]. Piagkou et al. [14] identified the high morphological variability of the medial orbital wall. The measurements were consistent between sides and sexes, while several differences were identified between nationalities [14].

Machine learning has increasingly transformed fields, especially in anatomy and medical sciences, offering powerful tools for analyzing complex biological structures and uncovering patterns that are often subtle or challenging to detect [15,16]. In anatomical studies, machine learning can identify key differences in various skeletal and soft tissue measurements, supporting applications from clinical diagnostics to treatment planning [17,18]. For example, by analyzing large anatomical datasets, machine learning models can distinguish sex-, age-, and even population-specific traits. This technology is precious in forensic science for cases where skeletal remains are incomplete, degraded, or lacking other identifying features.

When applied to precise anatomical measurements—such as those from the orbit of the skull—machine learning models can improve the accuracy of sex estimation and provide critical insights for forensic scientists. This capability allows for the reconstruction of identities from skeletal remains, even in challenging cases where traditional methods may fall short [19,20,21,22,23]. Moreover, as the field continues to evolve, the integration of machine learning with advanced image processing, scanning and measuring techniques, and automated feature extraction methods holds the potential to further expand forensic anthropology through the exploration of new biomarkers and correlations that will help experts make more reliable determinations and improve the overall efficiency of forensic investigations [24,25].

In the present study, we conducted a series of measurements on the human skull and employed a machine learning approach to evaluate their effectiveness in determining sex. Rather than aiming to develop a flawless classifier, our primary objective was to assess the discriminatory power of these features and identify those most contributing to classification accuracy. We also discuss the potential applications of this research within the field of forensic science.

## 2. Materials and Methods

One hundred and twenty (120) dried adult human skulls were used. Similar to previous studies, inclusion criteria were the good quality of the orbits and the known sex, while exclusion criteria were destroyed orbits and/or unknown sex [26]. Therefore, ninety-two (92) skulls were used. The sexes were distributed as follows: 35 males and 57 females. The age of the specimens was unknown. The sample was derived from the osteological collection of the Department of Anatomy (School of Medicine, National and Kapodistrian University of Athens).

**Figure 1:**
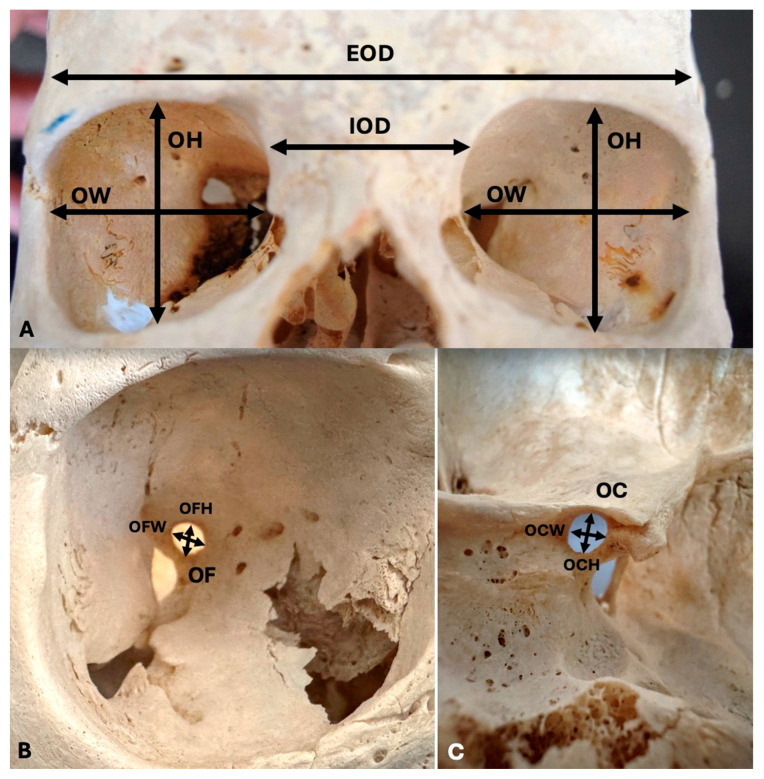
The orbital measurements of the current study. (**A**) Orbit height (OH), orbit width (OW), infraorbital distance (IOD), extraorbital distance (EOD); (**B**) optic canal (OC), (**C**) and optic foramen (OF).

The anatomical investigations were conducted by three anatomists (G.Tr., M.P., and G.Ts.), depicted in Figure 1. The following orbital measurements were performed: orbit width (OW), orbit height (OH), infraorbital distance (IOD), extraorbital distance (EOD), optic foramen width (OFW), optic foramen height (OFH), optic canal weight (OCW), and optic canal height (OCH). The orbit index (OI) was calculated by dividing the OH by the OW and multiplying it by 100. The optic canal index (OCI) was calculated by dividing the OCH by the OCW and multiplying it by 100. The optic foramen index (OFI) was calculated by dividing the OFH by the OFW and multiplying it by 100. An electronic caliper (Mitutoyo Corporation, Kawasaki-shi, Kanagawa, Japan) was used for the measurements. Each measurement was repeated twice with an accuracy of up to 0.1 mm.

Statistical analysis evaluated the differences and correlations between the orbital measurements. All analyses were conducted using the Python programming language and the SciPy package. Data preprocessing involved the application of z-normalization to replace outliers with z-scores exceeding 2.5 with the median value. Continuous variables were expressed as mean ± standard deviation (SD). For variables compared by sex, normality was assessed using the Shapiro–Wilk test, and homogeneity of variances was evaluated with Levene’s test. An independent samples *t*-test was performed when both assumptions were satisfied. The Mann–Whitney U test was employed when these assumptions were violated. The sexual dimorphism index (SDI) for each measurement was determined by dividing the mean value of males by the mean value of females. Comparisons of measurements concerning the sides were assessed using a paired t-test to determine if the differences between bilateral measurements met the normality assumption, evaluated by the Shapiro–Wilk test. The Wilcoxon Signed-Rank test was utilized if the normality assumption was not satisfied. Correlation analysis among variables was performed by computing the Pearson correlation coefficient when both variables exhibited a normal distribution, as verified by the Shapiro–Wilk test, and homoscedasticity, as assessed by Levene’s test. The Spearman correlation coefficient was computed when these assumptions were not met. Separate correlation analyses and matrices were generated for the overall variable measurements and the derived indexes from the original variables. A significance level of *p* < 0.05 was established for all statistical tests.

**Figure 2:**
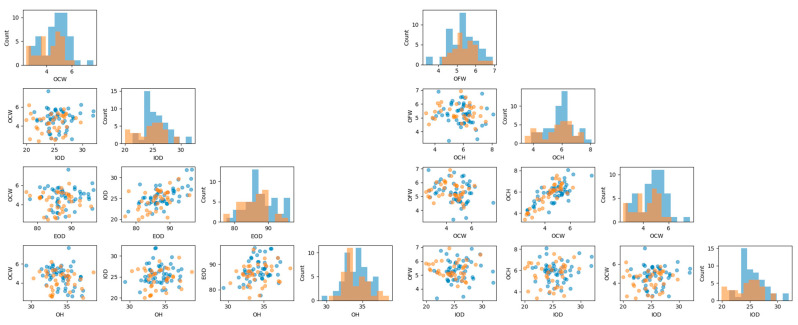
Scatter plots between features and bar plots of their frequencies. Relations of proportionality are noticeable (blue: female; orange: male).

After averaging the features from both sides into a single measurement, all orbital measurements were initially visualized to identify outliers and explore relationships among features (Figure 2). The Pandas library (version 1.5.3) was utilized for data manipulation and preprocessing, while NumPy (version 1.23.5) supported numerical operations.

All training procedures used a random forest classifier with 100 estimators, implemented via Scikit-learn (version 0.24.2). The dataset was split into an 80% training–validation set and a 20% test set, using stratified sampling to ensure equal sex representation.

An exhaustive search over all possible feature subsets was conducted on the training–validation set using 5-fold cross-validation to identify the top 5 feature subsets based on mean accuracy. Each of these top 5 subsets was then re-evaluated with another round of 5-fold cross-validation, training the model on each subset. The subset with the highest mean accuracy from this final evaluation was selected, and the model was retrained on the entire training set using this subset.

The final model was evaluated on the test set, with metrics such as precision, recall, F1 score, and ROC (receiver operating characteristic) AUC score calculated to assess classifier performance. Definitions for these metrics are provided in Equations (1)–(5).

(1) Accuracy

This metric represents the proportion of correct predictions (true positives and negatives) of all instances. It is calculated as:

(1) $$Accuracy=\frac{\left(True\;Positives+True\;Negatives\right)}{Total\;Instances}\;$$

(2) Precision

Precision measures the proportion of true positive predictions out of all the predicted positives. It is useful when minimizing false positives, which is essential. It is calculated as:

(2) $$Precision=\frac{TruePositives}{\left(True\;Positives+False\;Positives\right)}\;$$

(3) Recall

Recall (or sensitivity) measures the ability to identify all positive instances correctly. It is critical when it is important to catch as many positive cases as possible, even at the cost of more false positives. It is calculated as:

(3) $$Recall=\frac{True\;Positives}{\left(True\;Positives+False\;Negatives\right)}$$

(4) F1 Score

The F1 score is the harmonic mean of precision and recall. It balances the trade-off between the two and is particularly useful when both false positives and negatives are equally important. It is calculated as:

(4) $$F1\;Score=2\times \frac{\left(Precision\times Recall\right)}{\left(Precision+Recall\right)}$$

(5) ROC AUC (Area Under the Curve)

The ROC AUC score evaluates the model’s ability to distinguish between classes. It is calculated by plotting the true positive rate (recall) versus the false positive rate (FPR) and measuring the area under the curve. A higher AUC indicates better model performance.

(5) $$\left[\mathrm{AUC}=\int_{0}^{1}\mathrm{TPR}\left(t\right)\;d\left(\mathrm{FPR}\left(t\right)\right)\right]$$

Furthermore, feature importances for the selected subset were extracted and reported. In a Random Forest model, feature importance is determined by each feature’s contribution to reducing impurity—such as Gini impurity or entropy—averaged across the decision trees in the forest. Features that lead to more significant impurity reduction are considered more important. These importance scores are normalized to sum to one, meaning that the total importance across all features is scaled so that the sum equals one. This normalization allows for the easy comparison of feature contributions, highlighting the most influential features in the model’s decision-making process.

## 3. Results

The basic anatomical results are summarized in Table 1. Between the left and right measurements, there was no statistically significant result. The following measurements were statistically significant between sexes: the left OCW (*p* = 0.003), the EOD (*p* = 0.040), and the left OW (*p* = 0.010). All the other measurements were not significant between sexes (Table 1).

All measurements were correlated with each other, and the statistically significant results, which also fulfilled the correlation requirements, are further discussed. The analysis revealed significant correlations between EOD and IOD, as well as between OCH and OCW. EOD and IOD exhibited a strong significant correlation with a correlation coefficient of 0.5668 and a *p*-value of <0.001. Similarly, OCH and OCW demonstrated a significant correlation with a correlation coefficient of 0.7493 and a *p*-value of <0.001, highlighting a very strong association between these variables.

The feature selection process for the Random Forest classifier involved an exhaustive search over all possible feature subsets. The top five feature subsets based on mean accuracy during the cross-validation phase were identified as follows: subset: (‘OFH’, ‘OFW’, ‘IOD’, ‘EOD’, ‘OW’), mean accuracy: 0.6838; subset: (‘OCH’, ‘OCW’), mean accuracy: 0.6724; subset: (‘OFW’, ‘OCH’, ‘OCW’, ‘IOD’, ‘EOD’), mean accuracy: 0.6724; subset: (‘OFH’, ‘OCW’, ‘IOD’, ‘EOD’, ‘OW’), mean accuracy: 0.6714; subset: (‘IOD’, ‘EOD’), mean accuracy: 0.6705. The best subset of features selected for the Random Forest classifier included OW, IOD, EOD, OFH, and OFW. Using this subset, the final evaluation yielded a test accuracy of 0.68, a precision of 0.65, a recall of 0.70, an F1 score of 0.675, and an ROC AUC score of 0.72. Further analysis of feature importances within this optimal subset revealed that OW had the highest influence, with an importance score of 0.2429, followed by IOD at 0.2059, EOD at 0.1927, OFH at 0.1798, and OFW at 0.1787. These importance scores were normalized to sum to one, representing each feature’s proportional contribution to the model’s predictive capability. These results underscore the significance of feature selection in enhancing the model’s performance and provide valuable insights into the relative importance of each feature in the predictive model.

## 4. Discussion

In the present study, we performed multiple measurements of the human orbit and investigated their relationship and possible application for sex estimation. Only a few anatomical studies have used a machine learning approach [5,27,28]. According to the current results, the orbital structures can serve as possible measurements for sex estimation, which has implications for forensic science. Nevertheless, we depicted important correlations between some orbital measurements. The EOD–IOD and OCH–OCW were positively correlated. Similar correlations were observed by Packirisamy et al. [6]. We also evaluated if the orbit measurements correlated with the OC measurements; however, the results were insignificant. This relationship was not evaluated in the current literature, and further studies should be performed for these structures.

In the current study, the left OCW, the EOF, and the left OW were statistically significant between sexes. Packirisamy et al. [6] identified this difference for the left and right OW, left and right OH, EOD, and IOD in their study. They used these six measurements for the sex estimation. The left and right OH were the most reliable measurements, with 80.7% and 83.8% for the univariate analysis in the Saudi Arabian population [6]. The left and right OW was the second most reliable sex predictor, with accuracy rates of 79.4% in the Packirisamy et al. [6] study. Nevertheless, the accuracy rate for determining sex reached 92.1% [6]. In another recent study, Kresic et al. [11] used twelve orbital measurements and identified an overall accuracy of 73.45% in the Croatian population. The simple orbital measurements obtained an overall multivariate analysis that reached an accuracy rate of 73.2–92.1% [6,29]. The current study’s overall accuracy rate was 83%, which is close to the current literature. However, the simple orbit measurements are similar to the current research and the Packirisamy et al. [6] study, so more complicated models of the orbit region have been performed. Graillon et al. [1] investigated the sexual dimorphism of the orbital volume with three-dimensional reconstruction. Their sex estimation test had a precision of 77.3% [1]. Ajanovic et al. [12] performed a detailed geometric morphometric analysis of the shape and size of the orbits. Their results showed that sex estimation was possible with 86.33% accuracy for males and 88.69% for females. Thus, it is essential to mention that simple orbital measurements are as reliable as the most complex ones.

Except for the orbit structures, several other anatomical landmarks have been used for sex estimation. Baca et al. [30] performed eight measurements on the human pubic bone, including the pubic tubercle, superior and inferior pubic symphysis, lateral border, and pubic body height and width. They reported an overall accuracy rate of 95.49% [30]. Baca et al. [30] observed an accuracy rate between 90–95% in the current literature. Another reliable human structure that has been used for sex estimation is the sternum. The reported overall accuracy rate using the sternum surface area is 81.8–95% [31]. Nevertheless, Baban and Mohammad [32] evaluated the machine learning approach for sex estimation with human mandibles, and they observed a high accuracy of up to 90%. Recently, the pterion, an important neurosurgical landmark of the skull, was also evaluated. Uabundit et al. [27] identified an accuracy for sex prediction around 80%. Recently, the foramen magnum measurements depicted an accuracy level of 88.2% [3]. Moreover, the atlas vertebra was also examined for its applicability to sex estimation, with an acceptable level of accuracy (82.6%).

The use of a Random Forest classifier enabled the identification of multivariate interactions between features, capturing complex relationships that may not be apparent through traditional statistical methods. While only a subset of orbital measurements showed statistically significant differences between sexes, the multivariate approach allowed us to identify feature subsets that contributed to the predictive power of the model. The presence of OW in all the best subsets points towards the higher importance of this feature in sex estimation. Moreover, IOD appeared in most of the best feature subsets, implying a similar importance weight. Further, features such as EOD, OFH, and OFW were consistently included in the best subsets, indicating their combined importance.

We averaged the measurements from each side to ensure consistency and reduce dimensionality, as the differences between the left and right sides of the same gender were not statistically significant. This approach helps mitigate noise and variability from side-specific differences, capturing overall trends and patterns in the data. While some measurements were significantly different on one side, averaging ensures a balanced and comprehensive feature set, enhancing the model’s performance and generalizability. This method aims to create a feature set less prone to overfitting and more representative of the overall anatomical structure, as evidenced by our cross-validation results.

Despite achieving a test accuracy of 68%, with a precision of 0.65, indicating a moderate rate of false positives, the recall score of 0.70 reveals that 30% of the true positives were missed, which is a significant limitation for applications where detecting all positive cases is critical, such as forensic sex estimation. The AUC score of 0.72 further suggests a reasonable model performance in distinguishing between classes. These results suggest that orbital measurements could complement other anatomical markers in forensic applications, even though the current classifier performance indicates room for improvement.

Given the dataset size of 92 samples, with 35 males and 57 females, the imbalance in sample distribution likely contributed to the observed performance gaps. Imbalanced datasets can skew the learning process, as the model may become biased toward the majority class, leading to suboptimal performance in classifying the minority group. This issue underscores the critical need for more balanced datasets to ensure fair representation of both sexes and improve model generalization. Techniques such as data augmentation, resampling strategies, or class-specific weighting could be considered in future studies to mitigate this limitation.

Additionally, the decision to limit the number of estimators to 100 was made to prevent overfitting, as increasing the number of estimators could have led to excessive model complexity and poor generalization to new data. This highlights the inherent trade-off between model complexity and performance when working with small and imbalanced datasets. Together, these factors emphasize the challenges of building robust and generalizable machine learning models in forensic applications where data availability is often limited.

The main application of sex estimation is mainly for forensic science. In forensic anthropology, the pelvis can be a reliable and relatively quick method to estimate the sex of the human body. However, if the pelvic bone is destroyed or damaged, it is essential to have reliable alternatives. Thus, extensive research on various anatomical structures has been performed. The skull is a relatively dense and well-preserved structure of skeletal remains. Therefore, skull measurements could be an excellent alternative to the pelvis for forensics. Specifically, orbital measurements are a simple method that does not always require computed tomography analysis and three-dimensional reconstruction (3DR), such as the complex measurements proposed by recent studies.

The present study has several limitations that should be considered when interpreting the results. Firstly, the relatively small sample size (*n* = 92) may limit the generalizability of the findings. A larger and more diverse sample, particularly from different geographic regions or with varied demographic characteristics, would provide more accurate insights. In this study, the sample was limited to individuals from Athens, Greece, and further research incorporating samples from different nationalities and ethnic groups would be beneficial to understand the potential global applicability of the findings.

Additionally, while the study indicates the importance of specific anatomical measurements—such as OFW, OFC, OCW, and OCH—in sex estimation, the sample size and the imbalance in gender distribution (35 males and 57 females) may have influenced the statistical power of these results. Larger and more balanced datasets are crucial for confirming these preliminary findings and validating the selected features’ performance in more representative populations. The current imbalance in the sample likely contributed to performance gaps in the model, underscoring the importance of addressing class imbalances when training machine learning models.

## 5. Conclusions

The current study highlights the potential of orbital measurements as informative features for automated sex estimation in forensic contexts. While the significant correlations among various orbital parameters underscore their relevance, the observed performance metrics of the Random Forest classifier suggest that these measurements alone may not yet achieve reliable classification for practical application. These findings point to the need for further research to refine the discriminatory power of orbital measurements, explore complementary features, and evaluate their integration into broader forensic identification methodologies. Such advancements could ultimately enhance the accuracy and applicability of sex determination techniques in forensic science.

## Figures and Tables

**Table 1:** The orbital measurements are distributed according to sides and sexes. All results are presented as mean (standard deviation). OH—orbit height; OW—orbit weight; IOD—intraorbital distance; EOD—extraorbital distance; OCH—optic canal height; OCW—optic canal width; OFH—optic foramen height; OFW—optic foramen width.

Parameters	Males (*n* = 35)		Females (*n* = 57)	
	Left Side	Right Side	Left Side	Right Side
OH	34.11 (2.11)	33.74 (1.90)	33.96 (1.65)	34.21 (2.08)
OW	39.01 (2.54)	39.14 (2.77)	40.24 (1.97)	39.51 (2.12)
IOD	24.60 (2.41)		25.68 (2.26)	
EOD	85.77 (4.25)		87.68 (4.36)	
OCH	5.52 (1.34)	5.66 (1.12)	5.85 (1.14)	5.92 (0.89)
OCW	4.29 (1.04)	4.29 (1.04)	4.92 (0.82)	4.73 (1.10)
OFH	3.92 (0.58)	3.94 (0.78)	4.16 (0.73)	4.06 (0.85)
OFW	5.58 (0.72)	5.14 (0.70)	5.34 (1.03)	5.25 (0.83)

## Data Availability

The data are available to the corresponding author upon reasonable request.
